# Targeting the microRNA-34a as a Novel Therapeutic Strategy for Cardiovascular Diseases

**DOI:** 10.3389/fcvm.2021.784044

**Published:** 2022-01-27

**Authors:** Cun-Cun Hua, Xin-Ming Liu, Li-Rong Liang, Le-Feng Wang, Jiu-Chang Zhong

**Affiliations:** ^1^Heart Center and Beijing Key Laboratory of Hypertension, Beijing Chaoyang Hospital, Capital Medical University, Beijing, China; ^2^Department of Clinical Epidemiology and Tobacco Dependence Treatment Research, Beijing Institute of Respiratory Medicine, Beijing, China; ^3^Beijing Institute of Respiratory Medicine, Capital Medical University, Beijing, China

**Keywords:** microRNA-34a, cardiovascular fibrosis, apoptosis, myocardial remodeling, heart dysfunction

## Abstract

Cardiovascular diseases (CVDs) are still the main cause of morbidity and mortality worldwide and include a group of disorders varying from vasculature, myocardium, arrhythmias and cardiac development. MicroRNAs (miRs) are endogenous non-coding RNAs with 18–23 nucleotides that regulate gene expression. The miR-34 family, including miR-34a/b/c, plays a vital role in the regulation of myocardial physiology and pathophysiological processes. Recently, miR-34a has been implicated in cardiovascular fibrosis, dysfunction and related cardiovascular disorders as an essential regulator. Interestingly, there is a pivotal link among miR-34a, cardiovascular fibrosis, and Smad4/TGF-β1 signaling. Notably, both loss-of-function and gain-of-function approaches identified the critical roles of miR-34a in cardiovascular apoptosis, autophagy, inflammation, senescence and remodeling by modulating multifunctional signaling pathways. In this article, we focus on the current understanding of miR-34a in biogenesis, its biological effects and its implications for cardiac pathologies including myocardial infarction, heart failure, ischaemia reperfusion injury, cardiomyopathy, atherosclerosis, hypertension and atrial fibrillation. Thus, further understanding of the effects of miR-34a on cardiovascular diseases will aid the development of effective interventions. Targeting for miR-34a has emerged as a potential therapeutic target for cardiovascular dysfunction and related diseases.

## Introduction

Cardiovascular diseases (CVDs) are regarded as the leading cause of human death worldwide (1). Extensive studies have focused on the pathogenesis of CVDs, but the underlying pathophysiological mechanism is still not clear. Therefore, investigations into the molecular basis of CVDs may elucidate a new pathway for treatment in clinical practice. MicroRNAs (miRs) are endogenous non-coding RNAs with 18–23 nucleotides that regulate gene expression by binding complementary sequences in the 3'UTR of mRNAs. MiRs are indispensable in various biological processes and are involved in cell differentiation and proliferation, cell death, and metabolism (2). In the cardiovascular system, miRs regulate cellular pathways in cardiac fibrosis, apoptosis, inflammation, autophagy and senescence. Recent studies have identified that miRs are involved in multiple CVDs. The role of miR-34 family in regulating cardiac function is notable.

The miR-34 precursor family was discovered computationally and identified experimentally later. Three mature miRs, miR-34a, miR-34b, and miR-34c, are processed by two different precursors. The miR-34 family is transcribed from two distinct sets of genes located on chromosomes 1 and 11 (3). MiR-34a expression is higher than miR34b/c expression in most human organs and tissues except in the lungs (4). Currently, miR-34a expression is mainly regulated by epigenetic modification, transcriptional regulation and other molecular mechanisms (5). Notably, miR-34a has been elucidated to play crucial roles in diverse cardiac biological pathways including apoptosis, inflammation, autophagy, aging and fibrosis, which ultimately contribute to cardiac dysfunction, suggesting the therapeutic potential of targeting miR-34a in CVDs. This review article will discuss the biological effects of miR-34a and summarize the current understanding of miR-34a in the development and prevention of CVDs, including myocardial infarction (MI), heart failure (HF), ischaemia reperfusion (I/R) injury, cardiomyopathy, atherosclerosis, hypertension and atrial fibrillation (AF).

## Biogenesis and Biological Effects Of MIR-34A

**Figure 1:**
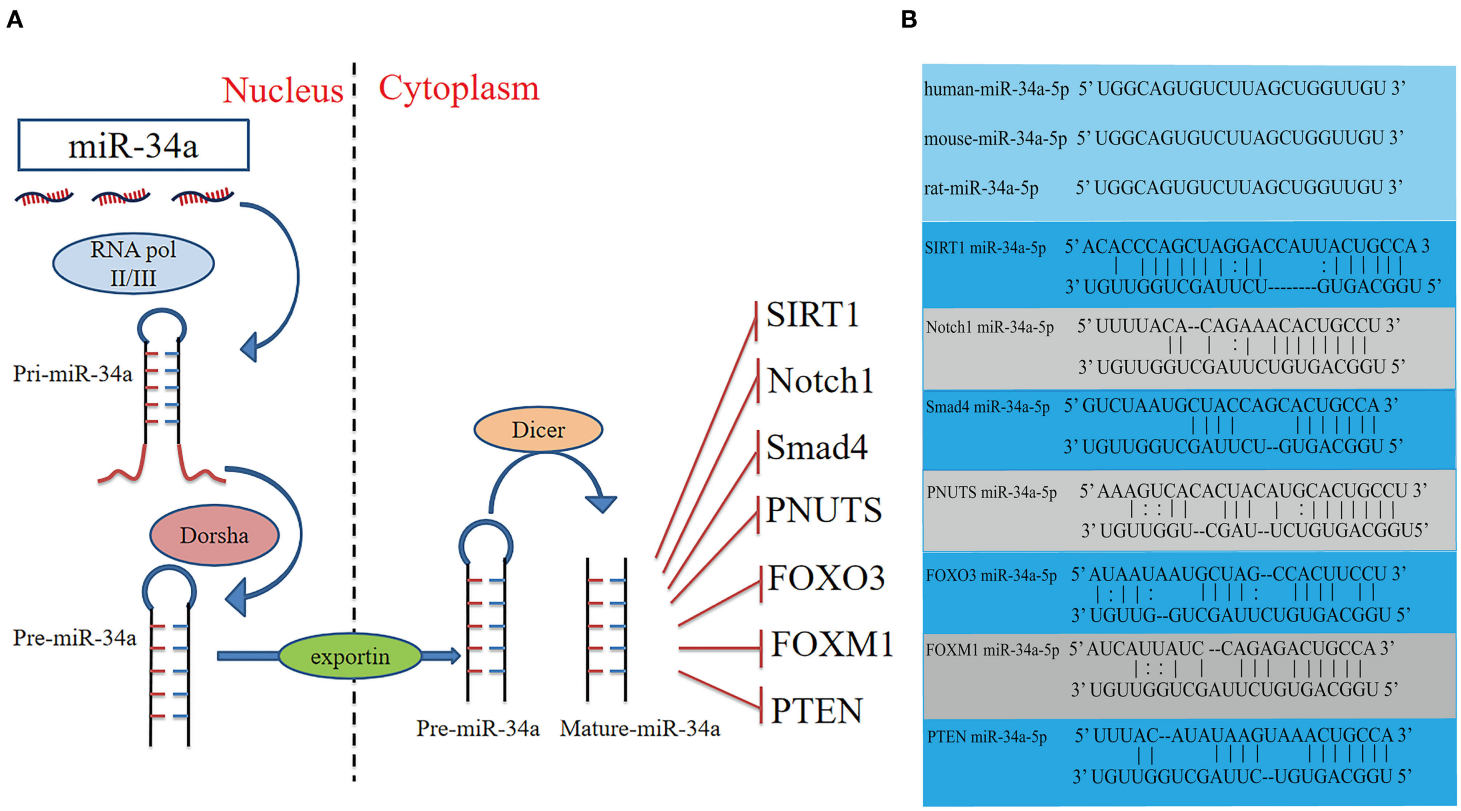
Schematic diagram of the activities of miR-34a, and potential binding sites between miR-34a and the target genes. **(A)** Schematic diagram of the activities, and target genes of miR-34a. **(B)** The sequence of miR-34a-5p is conserved in humans, mice and rats. The potential miR-34a-5p-binding sites of target genes. FOXM1, Forkhead box M1; FOXO3, Fork head box O3; PTEN, Phosphatase and tensin homologue; SIRT1, Silent information regulator 1.

MiRs are small non-coding RNAs which regulate gene expression by binding to complementary sequences in the 3'UTR of mRNAs. The miR-34 family was found to be increased in heart tissue from patients with cardiovascular diseases and encoded by two different genes (6). MiR-34a is encoded by its own transcript, while miR-34b and miR-34c are encoded by the same primary transcript (7). Similar to the biogenesis of all miRs, miR-34a is transcribed from the transcription start site in the nucleus by RNA polymerase II/III as a long hairpin molecule (pri-miR) and then cleaved by an RNase III Drosha to a ~70-nucleotide long stem-loop precursor (pre-miR). The pre-miR is transported from the nucleus to the cytoplasm and is further cleaved by an RNase III Dicer into 22-nucleotide long mature strands, which are consolidated into the RNA-induced silencing complex (Figure 1). This complex downregulates target transcripts by mRNA degradation or inhibition of translation (8, 9). The human miR-34a precursor is transcribed from chromosome 1 and maps to the distal region of chromosome 1p. It is encoded in the second exon of a gene located on chromosome 1p36.22 (10, 11). MiR-34a is involved in cellular diseases and is recognized as an essential regulator in cardiovascular diseases (12).

**Figure 2:**
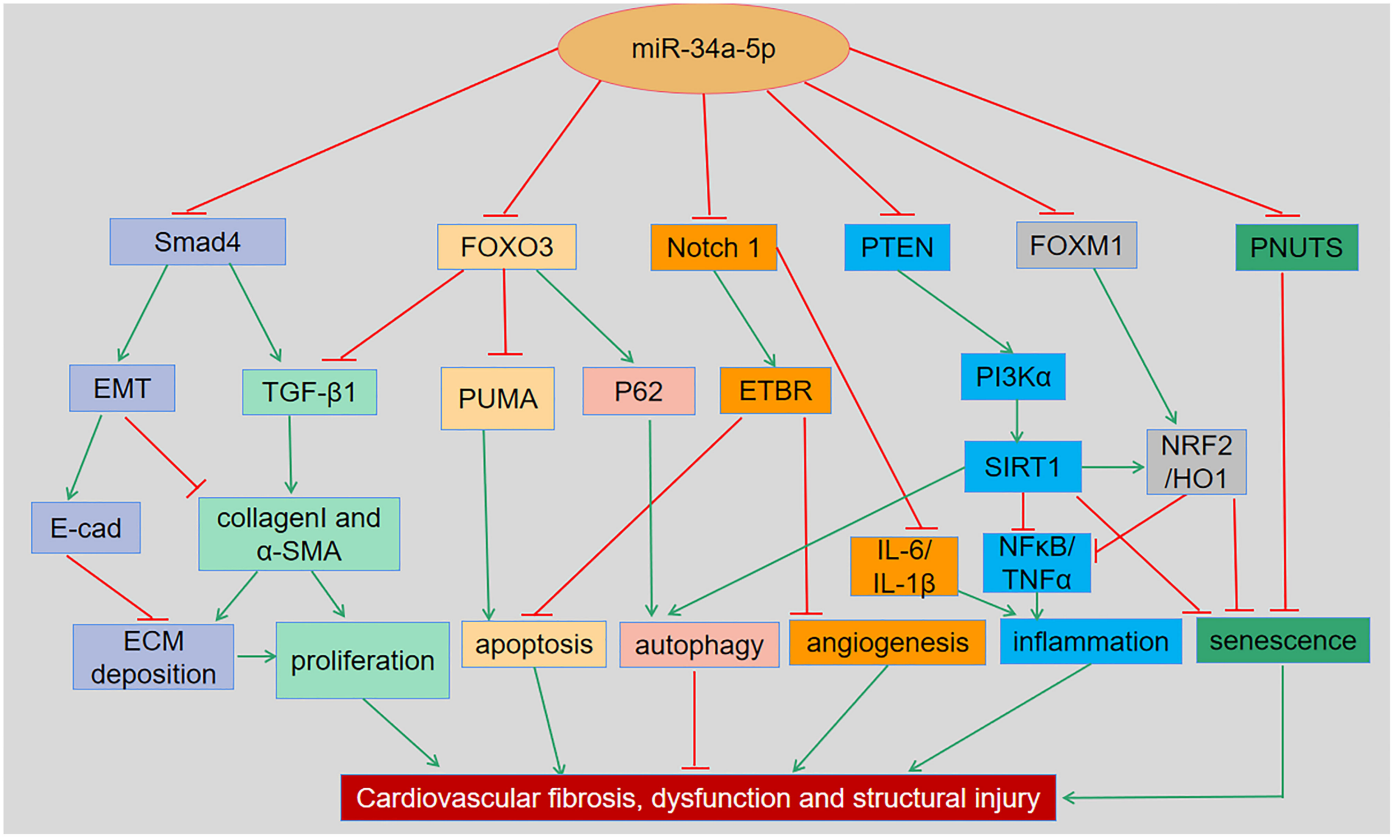
The regulatory roles and underlying mechanisms of miR-34a in cardiovascular dysfunction and related disorders. MiR-34a has been shown to regulate cellular apoptosis, autophagy, inflammation, senescence and remodeling in various cardiac and vascular tissues and cells through Smad4/TGF-β1, FOXO3/PUMA, Notch1/ETBR, PTEN/PI3K/SIRT1, and FOXM1/NRF2/HO-1 signaling pathways, respectively. EMT, Epithelial-mesenchymal transition; ECM, Extracellular matrix; ETBR, endothelial receptor type B; FOXM1, Forkhead box M1; FOXO3, Forkhead box O3; NRF2, Nuclear factor-E2-related factor 2; PI3K, Phosphatidylinositol 3-kinase; PNUTS, Phosphatase-1 nuclear targeting subunit; PTEN, Phosphatase and tensin homologue; PUMA, p53-up-regulated modulator of apoptosis; SIRT1, Silent information regulator 1; TGF-β1, Transforming growth factor β1.

It has been reported that overexpression of miR-34a promotes apoptosis of MI by inhibiting the activity of silent information regulator 1 (SIRT1) (13). Phosphatase and tensin homolog (PTEN), a negative regulator of PI3K signaling, was downregulated, while miR-34a expression was increased and mRNA of SIRT1 was decreased in oxidative stress-induced epithelial cells (Figure 2) (14). In addition, Notch1, Smad4, and fork head box O3 (FOXO3) were verified as miR-34a target genes (Figure 1) (11, 15), all of which have actions relevant to cardiovascular diseases. In congenital heart disease (CHD), overexpression of miR-34a induced the deduction of embryonic endocardial cells (ECCs) and acceleration of cell apoptosis, further increasing the risk of CHD targeting Notch1 through the Notch signaling pathway (Figure 2) (16). Interestingly, hypoxia-induced HIF1α accelerated angiogenesis of trophoblast cells through the Notch1/ endothelial receptor type B (ETBR) signaling pathway (17). ETBR antagonist increases apoptosis and susceptibility to an apoptotic agent in human pulmonary arterial smooth muscle cells, further promoting vascular remodeling in pulmonary artery hypertension (Figure 2) (18). Additionally, miR-34a enhanced cardiac fibrosis after MI *via* targeting Smad4 related with TGF-β1 signaling pathway while miR-34a inhibition ameliorated the above changes (Table 1; Figure 2) (19). PNUTS, also known as PPP1R10, was identified as a target gene of miR-34a, a novel, SIRT1 independent of the miR-34a signaling pathway, plays a vital role in many cellular processes including cell cycle progression, DNA damage responses (DDR), and apoptosis. It has been reported that PNUTS plays a major role in the anti-senescence effect of doxorubicin (DOXO)-induced H9C2 cell aging. More importantly, inhibition of miR-34a improves cardiac function during aging and post MI by reducing cell death and fibrosis by targeting PNUTS, which is an essential regulator of endogenous cardiac regeneration (Table 1; Figure 1) (20, 29). MiR-34a accelerated the progression of atherosclerosis by regulating FOXO3 expression. It was reported that FOXO3 plays a critical role in restraining oxidative damage in ox-LDL-induced endothelial cell injury *via* the miR-34a/SIRT1/FOXO3 signaling pathway (Table 1; Figure 2) (21). FOXO3 inhibited the transcription of pro-apoptotic gene p53 upregulated modulator of apoptosis (PUMA) to promote cell apoptosis (Figure 2) (30). It was identified that inhibition of miR-34a-5p alleviated lipopolysaccharide-induced human umbilical vein endothelial cells (HUVECs) injury by targeting FOXM1 through activating the Nuclear factor-E2-related factor 2 (NRF2)/HO-1 pathway (Figure 2) (31).

**Table 1:** The regulatory roles and underlying mechanisms of miR-34a in cardiovascular dysfunction and disorders.

**Experimental model**	**Experimental intervention**	**Effects**	**References**
MI mouse model	miR-34 family inhibitor	↑Vinculin, SIRT1, PNUTS, Notch1 ↑Bcl6, Sema4b ↓Left ventricular remodeling ↑Cardiac function	(6)
DOXO-induced myocardium	miR-34a inhibitor	↑Bcl2, SIRT1 ↓Apoptosis and senescence ↓Myocardial fibrosis and inflammation ↓Cardiac dysfunction	(12)
MI-induced rat myocardium	miR-34a mimic	↓Bcl2, SIRT1 ↑Bax ↑Apoptosis	(13)
MI rat model	miR-34a inhibitor	↓Apoptosis ↓TGF-β1 ↓CollagenI, α-SMA, ECM deposition	(19)
Cardiac fibroblast post MI	pre-miR-34a	↑Collagen I, α-SMA, fibronectin ↑TGF-β1, Smad4 ↑Proliferation, migration	(19)
Aged mouse	miR-34a inhibitor	↑PNUTS ↓Apoptosis, cell death ↓Cardiac hypertrophy ↑Cardiac contractile function	(20)
Mouse model of AMI	miR-34a inhibitor	↑PNUTS ↓Apoptosis ↓Fibrosis ↑Capillary density ↑Cardiac contractile function	(20)
ox-LDL-elicited HUVECs	miR-34a mimic	↑ROS, MDA ↓SOD, CAT, GSH,GPx, SIRT1, FOXO3a ↑Oxidative stress	(21)
Mouse CMECs	miR-34a mimic	↑IL-1β, IL-6 ↓Notch1, IL-10, VEGF, bFGF, HGF ↑Apoptosis ↓Cell viability and capillary-like structures of cells formation ability	(22)
ISO-induced rat myocardial fibrosis model	miR-34a inhibitor	↑c-Ski ↓Collagen I, α-SMA, TGF-β1 ↓Cardiac fibrosis ↓Cell proliferation	(23)
RBFox2 KO mice	miR-34a inhibitor	↑JPH2 ↓Anf ↑Cardiac function	(24)
H/R induced mouse cardiomyocytes	miR-34a inhibitor	↓Bax, caspase 3 ↑Bcl2 ↓ER stress and cell apoptosis	(25)
Apoe–/– mice fed with HFD	miR-34a inhibitor	↑Bcl2 ↓Bax, Caspase-3, and caspase-9	(26)
		↑Cell growth and viability ↓Atherosclerotic lesions	
IS-induced HUVECs and HA VSMCs		↑miR-34a, p53 ↓Notch-1, SIRT1, Wnt-1, Jag1, ↓Proliferation, migration ↑Apoptosis	(27)
Wire injury CAVS mice	miR-34a inhibitor	↑Notch1 ↓Runx2, α-SMA, VCAM1, Cadherin ↓LVEDD ↓Intraventricular septum diameter ↓Aortic velocity, aortic valve tissue area ↓Calcium deposition	(28)

*ALDH2, Aldehyde dehydrogenase 2; Anf, atrial natriuretic factor; ApoE–/–, Apolipoprotein E-defcient; α-SMA, α-smooth muscle actin; bFGF, Basic fibroblast growth factor; Bcl-2, B-cell lymphoma-2; Bax, Bcl-2 associated X protein; CAVS, Calcific aortic valve stenosis; CAT, catalase; CMECs, Cardiac microvascular endothelial cells; DOXO, doxorubicin; EF, ejection fraction; ER, Endoplasmic reticulum; FS, fractional shorting; GSH, glutathione; GPx, glutathione peroxidase; HCY, Homocystein; HGF, Human growth factor; HFD, high-fat diet; HUVECs, human umbilical vein endothelial cells; HAVSMCs, human aortic vascular smooth muscle cells; IL-1β, Interleukin 1 β; I/R, Ischemia/Reperfusion; IS, indoxyl sulfate; ISO, isoproterenol; LVEDD, left ventricular end-diastolic diameter; MI, Myocardial infarction; MDA, malondialdehyde; NF-kB, Nuclear factor kappa-B; ROS, reactive oxygen species; Runx2, Runt-related transcription factor 2; SIRT1, Silent information regulator 1; Sema4b, semaphorin 4B; SOD, superoxide dismutase; TGF-β, Transforming growth factor β; VCAM1, vascular cell adhesion molecule 1; VEGF, Vascular endothelial growth factor*.

Collectively, miR-34a plays a vital role in controlling cellular apoptosis, autophagy, inflammation, aging, fibrosis, and remodeling through Smad4/TGF-β1, FOXO3/PUMA, Notch1/ETBR, PTEN/PI3K/SIRT1 and FOXM1/NRF2/HO-1 signaling pathways.

## MiR-34a and Apoptosis in the Cardiovascular System

Apoptosis as one of the cell death programs contributes significantly to multiple cardiovascular diseases such as MI, myocardial ischaemia/reperfusion injury (MIRI), and HF with reduced ejection fraction (32). MiR-34a is expressed abundantly in multiple cardiovascular disease induced apoptosis. A large number of studies have revealed that miR-34a regulates cellular apoptosis through p53-dependent or independent pathway. In p53-dependent pathway, miR-34a-induced apoptosis partly relies on the wild-type p53 genes at least, and p53 is induced by miR-34a, suggesting that miR-34a can also function in a feedback loop to p53 (11). The tumor suppressor p53 is critical for tumor prevention through regulating cell-cycle checkpoints, while loss of p53 function tends to result in human cancer (33). More importantly, ablation of p53 contributed to cardiomyocyte apoptosis and spontaneous hypertrophy in HF by regulating the cardiac transcriptome. This suggests that p53 plays a pleiotropic role in cardiac tissue homeostasis and function (34). As a target of p53, miR-34a targets Bcl2 and SIRT1 to aggravate myocardial injury by exacerbating apoptosis and infarct size, decreasing left ventricular function (35). Intriguingly, SIRT1, one of the main targets of miR-34a, mediates cellular responses involving apoptosis, autophagy and mitochondrial biogenesis, which suppress the activity of p53 by posttranscriptional deacetylation of the p53 protein (Figure 1) (9, 36, 37). Additionally, research have found that p53 and miR-34a interact in a positive feedback loop and target SIRT1 involved in cell cycle progression, cellular senescence and apoptosis. The above results indicated that miR-34a may regulate cardiac homeostasis and cellular apoptosis by modulating p53 function. Interestingly, the research has founded that the ratio of miR-34a to SIRT1 was increased in older patients' myocardial tissue rather than in young patients post MI, implying severe apoptosis in older patients and explained why older patients recovered more slowly than young patients.

It has been accepted that miR-34a modulates cellular apoptosis in cardiovascular system in a manner dependent on cell types. Recent study reported that miR-34a promotes apoptosis and angiogenesis of cardiac microvascular endothelial cells (CMECs), and upregulates inflammatory cytokines, thus worsening CMECs damage through the Notch signaling pathway (Table 1) (22). In hypoxia-induced cardiomyocytes, the expression of miR-34a-5p was increased while miR-34a-5p knockdown ameliorated cardiomyocyte apoptosis and cardiac dysfunction by upregulating ZEB1 and anti-apoptotic proteins such as aldehyde dehydrogenase 2 (38). In a diabetic cardiomyopathy rat model, granulocyte-colony stimulating factor ameliorated diastolic dysfunction and reduced cardiomyocyte apoptosis through downregulation of miR-34a and upregulation of Bcl2 (39). However, the effect of inhibition of miR-34a was not observed in cardiac progenitor cells, which showed reduced proliferation (39). Suppression of miR-34a activity may have diverse effects depending on cell types, thus it is necessary to eliminate off-target effects by introducing miR-based therapy.

The Notch signaling pathway is involved in processes related to cell differentiation, proliferation, apoptosis and survival. In a rat model of CHD, miR-34a targeted Notch1, improved the expression of Jagged1, Hes1, and Hey2, promoted the apoptosis rate and decreased the proliferation rate (Figure 1) (16). Similar to MIRI-induced microvascular injury, inhibition of miR-34a alleviates brain tissue damage and neuronal apoptosis by activating the Notch1/HIF-1α signaling pathway in a cerebral I/R-induced rat model (40). Additionally, adenosine deaminases acting on RNA 2 (ADAR2), an enzyme acting on double-stranded RNAs, promoted cardiomyocyte proliferation and inhibited DOXO-induced cell apoptosis *via* the ADAR2/miR-34a regulation axis, which involves the RNA editing activity of ADAR2 (41). These findings indicated that miR-34a promoted cardiomyocyte apoptosis and aggravated myocardial injury through different mechanisms.

## MiR-34a and Autophagy in the Cardiovascular System

Autophagy plays a vital role in the development of various cardiovascular diseases (42). It has been reported that miR-34a suppresses autophagy in cancer cells (43). However, autophagy regulated by miR-34a has also been demonstrated in cardiovascular disease. The inhibition of miR-34a enhanced cardiomyocyte autophagy and further ameliorated cardiac function in diabetic cardiomyopathy (44). In addition, miR-34a has been shown to inhibit autophagy after cardiac I/R injury by regulating TNF-α expression, thereby ameliorating myocardial damage (45). Interestingly, miR-34a-5p was found to be elevated in human coronary artery endothelial cells (HCAECs) exposed to chronic intermittent hypoxia (CIH), further increasing autophagy-related proteins such as Beclin and LC3. These results indicated that miR-34a-5p contributed to CIH-induced HCAECs autophagy through Bcl2/Beclin signaling pathways. MiR-34a-5p has been recognized as a potential therapeutic target for autophagy induced by CIH which leads to CVDs (46). Accordingly, miR-34a inhibition improves cardiac function by regulating protective autophagy in CVDs.

## MiR-34a and Cardiovascular Oxidative Stress and Inflammation

MiR-34a is a driver of vascular and systemic inflammation, which promotes the progression of inflammation related disease (47). Overexpression of miR-34a enhanced radiation-induced oxidative stress in cardiomyocyte, while macrophage migration inhibition reduced cellular reactive oxygen species (ROS) through the miR-34a/SIRT1 signaling pathway (48). More importantly, inhibition of miR-34a mitigated oxidative stress induced by H_2_O_2_ to protect vascular endothelial cells by targeting SIRT1 (49). The role of inflammation in MIRI is still controversial. Enhancement of circulating monocyte levels and attenuated repair capacity after MI have been found in apolipoprotein E-null mice (50). In an additional study, anti-inflammatory therapy was shown to reduce the number of cardiac adverse events post MI (51). MI and MIRI have been shown to cause a burst of ROS accumulation. Down-regulation of miR-34a alleviates the inflammatory response induced by renal I/R by promoting Kruppel-like factor 4 (KLF4) levels in rats (52). Similarly, miR-34a is involved in cancer immunotherapy-mediated myocardial inflammation. Cancer immune therapy has become a well-known treatment for some cancers, but immune checkpoint inhibitors (ICIs) more often lead to cardiac injury. It has been reported that inhibition of miR-34a attenuates cardiomyocyte inflammation induced by ICIs targeting KLF4 which has a cardioprotective effect. This result indicated that targeting the miR-34a/KLF4 signaling pathway may be an effective approach for patients who experience ICISs therapy-induced cardiac injury (53). In general, treatment with the miR-34a inhibitor may alleviate cardiomyocyte inflammation and oxidative stress. However, further studies are needed to identify the role of miR-34 in the treatment of inflammation-mediated MI and MIRI.

## MiR-34a and Cardiovascular Senescence

Age-related cardiac disease is driven by multiple molecular mechanisms such as telomere shortening, DDR, accumulation of somatic mutations, epigenetic changes, and alterations in non-coding RNAs regulating gene expression (54). Radiotherapy increases the survival of various cancer patients but leads to cardiac dysfunction, including cardiomyocyte senescence. A recent study demonstrated that the expression of miR-34a was obviously increased in the radiation-induced cardiac dysfunction to exert a pro-senescence effect on cardiomyocytes by regulating SIRT1 (Figure 2). Additionally, overexpression of miR-34a leads to a reduction in both the mRNA and protein levels of SIRT1/6, which are putative anti-aging enzymes, thereby promoting aging responses under oxidative stress (14). In the DOXO treated rat model, silencing of miR-34a ameliorated DOXO-induced myocardial senescence and protected cardiac function and integrity by targeting Bcl2 and SIRT1 (Table 1) (12). Suppression of miR-34a does not solely inhibit cardiomyocyte senescence but likewise plays a regulatory role in vascular senescence. A recent review generalized the role of miR-34a in vascular aging and senescence, demonstrating that miR-34a promoted vascular aging through targeting SIRT1 or regulating vascular cell adhesion protein1 and intercellular adhesion molecule 1 at least partially (55). More importantly, activation of FOXO3 promoted stress resistance in differentiated human vascular endothelial and smooth muscle cells which related to cellular aging (56). This may provide additional evidence that miR-34a promotes vascular aging and senescence by targeting FOXO3. The above results show that inhibition of miR-34a alleviated cellular senescence and further improved cardiac and vascular function, which may provide a new approach for protecting against radiotherapy-induced cardiac injury and HF.

## MiR-34a and Cardiovascular Fibrotic Remodeling

Myocardial inflammation and oxidative stress play critical roles in cardiac fibrosis and remodeling (57). It has been well-documented that miR-34a contributes to cardiac fibrosis and aging (58). The important pathological reactions that affect ventricular remodeling post MI encompass myocardial apoptosis, myocardial pathological hypertrophy, cellular senescence, and extracellular matrix metabolism disorder (59). It was identified that miR-34a could modulate TGF-β1 induced myocardial proliferation and ECM deposition of rat cardiac fibroblasts by targeting c-ski. MiR-34a inhibitor alleviated rat cardiomyocyte proliferation and ECM deposition induced by TGF-β1 (Table 1; Figure 2) (23). A recent study indicated that sevoflurane preconditioning inhibited cardiomyocyte apoptosis and reduced cardiac injury in hypoxia/reoxygenation (H/R) stimulated H9C2 cells. MiR-34a-5p attenuated the protective effect of preconditioning with sevoflurane in H/R induced H9C2 cells *via* targeting syntaxin 1A (60). MiR-34a inhibition has been shown to alleviate ventricular remodeling and improve cardiac function after myocardial infarction in animal models (20). Inhibition of miR-34a-5p promoted adipose-derived stem cell proliferation and migration to protect the myocardium against MI damage by activating the expression of c1q/tumor necrosis factor related protein-9 (CTRP9) (61). Anti-miR-34 attenuates MI-induced ventricular remodeling and atrial enlargement and improves cardiac function by targeting Notch1 (Table 1) (6). Furthermore, miR-34a plays an important role in regulating vascular smooth muscle cell functions and neointima hyperplasia, indicating its potential treatment application for vascular diseases (62). The expression of miR-34a decreased in hypoxia-induced human pulmonary artery smooth muscle cells and the reduction in miR-34a increased proliferation, conversely, overexpression of miR-34a reversed the above effects by targeting platelet-derived growth factor receptor alpha (PDGFRA). PDGFRA is an important regulator in pulmonary artery hypertension mediates cell proliferation and vascular remolding (63). In this regard, in primary hypertension, miR-34a has not been reported to regulate vascular remolding *via* targeting PDGFRA. These miR-34a-mediated apoptosis, proliferation and necrosis ultimately contribute to cardiac remodeling (64). Contrary to most studies, a recent report shows that miR-34a ameliorates myocardial fibrosis by reducing type I collagen production, cell viability, and migration and improves cell function in diabetic cardiomyopathy (65). In general, miR-34a inhibition alleviated cardiac and vascular remodeling post ischaemic disease. Glucose metabolism-related cardiac fibrosis may result in differential targeting effects of miR-34a.

Taken together, these findings show that inhibition of miR-34a exerts a protective effect on the pathophysiology of cardiovascular diseases and is a potential therapeutic strategy for improving cardiac function.

## Roles of miR-34a in MI and HF

MI remains a leading cause of morbidity and mortality worldwide. The infarcted heart is characterized by a loss of cells from death and myocardial fibrotic scarring. Cardiac hypertrophy and fibrosis largely lead to thickening and stiffening of the ventricular wall, further contributing to cardiac remodeling that ultimately results in cardiac dysfunction and subsequent heart failure (66, 67). MiR-34a is involved in a variety of pathological process of AMI through regulating cardiomyocyte proliferation, migration, apoptosis and remodeling. The expression of miR-34a increased in an MI rat model, leading to myocardial apoptosis, fibrosis and cardiac dysfunction through the Wnt/β-catenin pathway, indicating that miR-34a may be a potential therapeutic target for MI (68). In this regard, a recent study demonstrated that miR-34a was highly expressed in the heart tissue of AMI, which promoted cell apoptosis and oxidative stress. The results indicated that inhibition of miR-34a attenuated myocardial injury by multiple gene regulation of cellular phenotypes (69). Recent studies have shown that some mature miRs are also modified by m6A/m5C methylation, and that these modified mature miRs are significantly differentially expressed among cancer tissues (70). Similarly in the cardiovascular system, methylation of miR-34a promoted by Bcl-6 has a cardioprotective effect on AMI *via* the EZH2/miR-34a/CTRP9 axis (69). In the early stage of AMI, prevention of heart failure and left ventricular remodeling are closely related to the prognosis of patients, and left ventricular remodeling after AMI is associated with poor prognosis (68). It is becoming increasingly clear that miRs are promising predictors for cardiovascular pathological processes, including MI, myocardial remodeling and progression to heart failure. Long-term remodeling is associated with increased risk of cardiovascular death. Thus, early identification of ventricular remodeling is essential for clinical medicine (71). MiR-34a is upregulated and associated with left ventricular diastolic dimension in the convalescent stage of AMI patients who survive AMI but ultimately experience HF within 1 year (72). In addition, miR-34a exacerbated cardiac fibrosis and left ventricular dysfunction in the early stage of HF post AMI. It was demonstrated that miR-34a serves as an available predictor for future progression of HF post AMI (73). The results implied that miR-34a is an important biomarker for predicting left ventricular (LV) remodeling after AMI. Elevated miR-34a levels impair myocardial excitation contraction coupling which further leads to cardiac dysfunction through targeting Jph2. Importantly, it was demonstrated that intervention of this pathway with miR-34a antagonist significantly alleviated the heart failure in RBFox2 KO mice (24). These results indicated that inhibition of miR-34a plays a key role in controlling cell viability and cardiomyocyte apoptosis further improving cardiac function in MI and HF.

## Roles of MiR-34a in I/R Injury

Cardiovascular disease accounts for more than 40% of pathological occurrences, and the mortality and prevalence have increased in recent years (74). Ischaemic heart disease is a main cause of cardiovascular diseases, which mostly leads to MI and cardiac dysfunction (75, 76). The roles of miRs in I/R injury are mediated through the regulation of essential signaling pathways involved in necrosis, apoptosis, oxidative stress, inflammation, fibrosis and angiogenesis (77). It has been demonstrated that overexpression of miR-34a aggravates myocardial injury by increasing cell apoptosis and infarct size and decreasing LV function, while miR-34a suppression ameliorates myocardial I/R injury (35). Consistent with the above results, a recent study showed that miR-34a-5p inhibitor attenuated myocardial I/R injury by alleviating apoptosis rate and reducing infarct size and ROS accumulation through targeting Notch receptor 1 signaling (76). Crocin, one of the active components of saffron, has been identified to suppress I/R-induced endoplasmic reticulum stress and cardiomyocyte apoptosis through downregulation of miR-34a and activation of the SIRT1/NRF2 pathway (Table 1; Figure 2) (25). Accordingly, miR-34a inhibition protected cardiomyocytes against I/R injury, and future studies should be investigated the efficacy of treatment with miR-34a inhibitor in I/R patients and the underlying mechanisms.

## Roles of MiR-34a in Cardiomyopathy

Cardiomyopathy is a disease that negatively affects cardiac function and can be classified into primary and secondary categories. Hypertrophic cardiomyopathy is the most common primary cardiomyopathy. More importantly, diabetes-induced cardiac dysfunction has been increasingly attracted worldwide; however, reports of mechanism of diabetic cardiomyopathy are limited (78–80). MiR-34a increased high glucose-induced inflammation, oxidative stress and cell death by targeting SIRT1 in H9C2 cells (81). MiR-34a was overexpressed to promote apoptosis in the diabetic heart and glucose control did not change the expression of miR-34a (82). In addition, transfection with miR-34a inhibitor significantly reduced cell apoptosis in diabetic cardiomyopathy. Treatment with dihydromyricetin decreased miR-34a expression further alleviating autophagy in high glucose-induced cardiomyocytes and in the heart tissue of diabetic mice to ameliorate cardiac dysfunction (44). Hypertension-induced hypertrophic cardiomyopathy increases the occurrence of cardiovascular events. MiR-34a has been shown to be a negative regulator in hypertrophic cardiomyopathy to aggravate myocardial cell inflammation and immune responses through TGF-β/Smad signaling pathway (83). In particular, silencing miR-34a alone serves a protective role in moderate cardiomyopathy while anti-miR-34a does not alter the severe hypertrophic cardiomyopathy phenotype (84).

## Roles of MiR-34a in Atherosclerosis

The development of atherosclerosis is facilitated by the presence of concomitant risk factors, encompassing dyslipidaemial, leading to a chronic inflammatory reaction in the vessel wall. Endothelial cells (ECs) and vascular smooth muscle cells (VSMCs) are also involved in the development of atherosclerotic vascular disease (85). Macrophages play an important role in the pathogenesis of atherosclerosis by regulating inflammatory response and foam cell formation. MiR-34a regulated cholesterol effusion and inflammation in macrophages, reversed cholesterol transport, and promoted atherosclerosis by coordinating regulation of several genes related to the pathogenesis of atherosclerosis. Importantly, the inhibition of miR-34a significantly prevented the progression of atherosclerosis and reversed atherosclerosis *via* multiple signaling and different cell types (26, 85). In light of high level of miR-34a in vascular aging, it induces senescence and the acquisition of a senescence associated secretory phenotype in VSMCs and ECs. MiR-34a has been reported to trigger ECs aging through repression of SIRT1, whereas antagonism of miR-34a reduced ECs apoptosis and the development of atherosclerosis in ApoE^−/−^ mice (26, 86, 87). Interestingly, a recent study indicated that miR-34a/SIRT1/FOXO3 may play a vital role in reversing ox-LDL-induced oxidative stress in HUVECs by genistein (Figure 2) (21). Indoxyl sulfate can promote the process of atherosclerosis through upregulating miR-34a expression and inhibiting the Notch signaling pathway (27). The miR-34a/HNF4α pathway may play a critical role in the pathogenesis of non-alcoholic fatty liver disease and regulate plasma lipid and lipoprotein metabolism. It was identified that miR-34a reduced the development of atherosclerosis in ApoE^−/−^ mice by inhibiting HNF4α expression (Table 1) (26). Furthermore, a recent study suggested that miR-34a may be a promising target for the treatment of atherosclerosis as prevents cell growth and promotes apoptosis in atherosclerosis by regulating Bcl2, while deletion of miR-34a ameliorates atherosclerotic lesions in high-fat diet induced ApoE^−/−^ mice (87). Interestingly, miRs in plasma are emerging as novel biomarkers for predicting multiple diseases. High level of miR-34a was related to coronary artery disease independently [OR [95% CI: 3.87 (1.56-9.56)]; *P* = 0.003] and the increased level was associated with expression of SIRT1, JAG1,CTNNB1, ATF1, and Notch2 inversely (88). Thus, miR-34a plays a central role by promoting inflammation, apoptosis and senescence in macrophages, ECs and VAMCs to exacerbated atherosclerosis progression.

## Roles of MiR-34a in Hypertension

Primary hypertension is a common chronic disease that usually causes other health complications, such as coronary artery disease, stroke, renal failure and heart failure, and poses a great threat to human health (89, 90). Currently, the relationship between miR-34a and hypertension is relatively limited (91). Research has shown that miR-34a upregulated in the peripheral blood of patients with hypertension, and that upregulated miR-34a may promote vascular endothelial injury by targeting TIGF2 (89). The elevating miR-34a expression in hypertension increased the risk of vascular disease and related cardiovascular events. In hypertension-induced renal injury, urinary SIRT1 expression was decreased by miR-34a, indicating that SIRT1 is a potential method to evaluate renal injury in hypertensive patients (92). In contrary, a recent study showed that miR-34a reduces urinary microalbumin content in hypertensive mice and protects renal function targeting plasminogen activator inhibitor. The inhibition of miR-34a increased the levels of urinary microalbumin content and ACE protein, which contribute to renal damage in hypertensive mice (91). In general, miR-34a plays a critical role not only in hypertension but also in hypertension-related complications. The potential mechanisms between miR-34a and hypertension need further exploration.

## Roles of MiR-34a in AF

AF is the most common symptomatic cardiac arrhythmia in clinical medicine and shows an increasing prevalence with age. AF leads substantially to morbidity and mortality in the world (93). AF was divided into three groups at three durations (duration since diagnosis of AF). The 12-month and 24-month groups exhibited upregulated expression levels of miR-34a-5p (94). MiR-34a plays a critical role in promoting atrial electrical remodeling and the development of AF by inhibiting the expression of Ank-B (93). The present study revealed several novel persistent AF-associated miRs, including miR-34a-5p, which might target calcineurin1 and protein phosphatase three regulatory subunit B alpha to regulate persistent AF through the calcineurin-nuclear factor of activated T cells signaling pathway (95). These results provide valuable insights into the underlying mechanism of AF. The above results indicated that miR-34a may be a promising clinical diagnostic tool in the early stage of AF.

## Anti-MiR-34a Therapy in CVDs

It is well-known that miR-34a is a tumor suppressive miR in various tumor diseases. MiR-34a has been identified to have therapeutic potential for cancer. A recent study indicated that co-delivery 5-fluorouracil and miR-34a mimic improved the apoptotic effect and inhibited the metastasis of colorectal cancer cells (96). Further study demonstrated that pre-miR-34a transfection suppressed the expression of target genes, induced apoptosis and decreased the proliferation rate in gastric cancer (97). More importantly, this review showed that miR-34a plays a negative role in the progression of CVDs. Transfection with bone marrow-derived molecular cells promoted the release of IGF, which inhibited the expression of pre-miR-34a, thereby reducing cardiomyocyte apoptosis (98). Additionally, in calcific aortic valve stenosis model mice, locked nucleic acid-modified oligonucleotide (LNA)-miR-34a inhibitor attenuated the development of aortic valve stenosis and calcification (Table 1) (28). Inhibition of the miR-34 family attenuates pathological cardiac remodeling and atrial enlargement post AMI. Of interest, anti-miR-34a treatment ameliorated cardiac dysfunction by improving the cardiac molecular profile in females compared with males. More importantly, chronic administration of LNA-anti-miR-34 had no adverse effects on heart tissue morphology (99). The anti-miR-34 family, not anti-miR-34a only, shows a stronger therapeutic effect in cardiovascular disease, possibly due to regulating miRs and mRNA targets (100). Collectively, anti-miR-34a therapy is critical for CVDs and it is essential to explore the potential benefits of miR-34a inhibitor treatment in CVDs.

## Conclusion and Prospection

MiR-34a plays a vital role in the process of cardiovascular diseases. Recently, clinical and experimental studies have demonstrated that miR-34a plays a crucial role in diverse cardiac biological pathways that induce cardiovascular injury and dysfunction. MiR-34a overexpression has been revealed to blunt autophagy and promote cardiovascular apoptosis, inflammation, fibrosis, remodeling, aging and heart dysfunction. More importantly, the inhibition of miR-34a has been shown to exert pro-autophagic, anti-apoptotic, anti-oxidant, and anti-fibrotic effects in the cardiovascular system. This review systematically outlined the regulatory roles of miR-34a in cardiovascular dysfunction and provided evidence to support miR-34a as a potential therapeutic target for the treatment of cardiovascular diseases. More preclinical and clinical trials are needed to identify the potential benefits and side effects of anti-miR-34a therapy in cardiovascular diseases.

## Author Contributions

C-CH and X-ML drafted the manuscript. L-RL revised the manuscript. L-FW and J-CZ designed and provided guidance for the manuscript. All authors have read and approved the final manuscript.

## Funding

This work was supported by the General Program and the National Major Research Plan Training Program of the National Natural Science Foundation of China [92168117; 81770253; 91849111 and 81370362] and Talent project of Beijing Chaoyang Hospital Affiliated to Capital Medical University.

## Conflict of Interest

The authors declare that the research was conducted in the absence of any commercial or financial relationships that could be construed as a potential conflict of interest.

## Publisher's Note

All claims expressed in this article are solely those of the authors and do not necessarily represent those of their affiliated organizations, or those of the publisher, the editors and the reviewers. Any product that may be evaluated in this article, or claim that may be made by its manufacturer, is not guaranteed or endorsed by the publisher.
